# Characterization of oral bacterial and fungal microbiome in recovered COVID-19 patients

**DOI:** 10.1186/s12866-023-02872-3

**Published:** 2023-05-09

**Authors:** Nana Wei, Guangqi Zhu, Tingxiao Zhao, Yan Wang, Haifei Lou, Haoxuan Li, Zhejuan Yang, Zheen Zhang, Qiujing Wang, Mingfang Han, Zhibing Lin, Shibo Li

**Affiliations:** 1 Key Laboratory of Adolescent Health Assessment and Exercise Intervention of Ministry of Education, East China Normal University, Shanghai, 200241 China; 2 Department of Infectious Disease, Zhoushan Hospital, Wenzhou Medical University, Zhoushan, 316021 China; 3 Department of Infectious Disease, Zhejiang University Zhoushan Hospital, Zhoushan, 316021 China; 4 Wenzhou-Kean University, Wenzhou, 325060 China; 5 Shanghai Key Laboratory of Veterinary Biotechnology, School of Agriculture and Biology, Shanghai Jiao Tong University, Shanghai, 200240 China

**Keywords:** COVID-19, Oral bacteria, Oral fungi, Recovered patients, Virus clearance

## Abstract

COVID-19 has emerged as a global pandemic, challenging the world’s economic and health systems. Human oral microbiota comprises the second largest microbial community after the gut microbiota and is closely related to respiratory tract infections; however, oral microbiomes of patients who have recovered from COVID-19 have not yet been thoroughly studied. Herein, we compared the oral bacterial and fungal microbiota after clearance of SARS-CoV-2 in 23 COVID-19 recovered patients to those of 29 healthy individuals. Our results showed that both bacterial and fungal diversity were nearly normalized in recovered patients. The relative abundance of some specific bacteria and fungi, primarily opportunistic pathogens, decreased in recovered patients (RPs), while the abundance of butyrate-producing organisms increased in these patients. Moreover, these differences were still present for some organisms at 12 months after recovery, indicating the need for long-term monitoring of COVID-19 patients after virus clearance.

**Supplementary Information:**

The online version contains supplementary material available at 10.1186/s12866-023-02872-3.

## Introduction

COVID-19, which is caused by the novel coronavirus severe acute respiratory syndrome coronavirus 2 (SARS-CoV-2), has spread rapidly worldwide and poses a serious threat to public health globally [1]. SARS-CoV-2 transmission occurs primarily via respiratory transmission or direct contact with the virus [2]. Patients with COVID-19 show variable disease symptoms and severity. Although most infections result in no or mild symptoms, virus infection can lead to respiratory failure, organ dysfunction, and even death in some patients [3]. Old age, co-morbidity, tumors, and immunodeficiencies are risk factors connected to COVID-19 severity [4].

Previous studies have demonstrated that microbiomes in the body are crucial to development and maintenance of immune homeostasis. The oral cavity has the second largest microbiome after the gut in the human body [5]. Previous studies have confirmed that oral microbiota play significant roles in the pathogenesis of many infectious diseases [6]. For example, alterations in the oral microbiome are related to the onset and outcome of HIV [7] and HBV [8]. A retrospective cohort study indicated that the risk factors for COVID-19 are also heavily implicated in the dysbiosis of the oral microbiome [9]. Many studies have reported that oral hygiene interventions and professional care could reduce the progression of respiratory diseases, and improve clinical outcomes [10, 11]. During large outbreaks of SARS-CoV-2, numerous patients with COVID-19 have often exhibited complex co-infections with other pathogens, some of which originate from the oral cavity. However, the link between the oral microbiome and SARS-CoV-2 is not well understood.

The human oral microbiome has a complex composition that includes bacteria, fungi, viruses, and protozoa. Because oral fungi are relatively rare and many fungal species are uncultivable using current methods, the fungal microbiota found in the oral microbiome (also known as the mycobiome) have remained relatively unexplored, compared to the bacterial microbiota. However, novel metagenomic studies have shown the normal oral microbiome contains more fungal species than previously thought [12]. Moreover, there is evidence that fungi play important roles in the development of a robust host immune system [13, 14].

Previous studies have also reported that numerous viral pneumonia cases including COVID-19, showed bacterial–fungal coinfection, which may influence disease progression and outcome [15–17]. Li et al. [18] reported the changes in the bacterial community composition of patients with COVID-19. However, the oral bacterial profile of recovered patients and alternations in oral fungal communities following infection were not evaluated. Thus, in this study, we investigated the profile of oral bacterial microbiota and mycobiomes using Illumina 16 S rRNA and ITS sequencing.

## Materials and methods

### Study subject and sample collection

A total of 23 patients that had recovered from COVID-19 (RPs) and 29 healthy individuals (HCs) were included in this study. The lifestyle (including physical activity, schedule and actual sleep time), and diet habits (including food preference, appetite and body weight) of all the recovered patients had no significant changes during one year follow-up of study. Subjects with smoking habits were excluded from the study. Samples from RPs were collected in Zhoushan Hospital, China. All recovered patients had been COVID-19 free for more than 12 months with no significant clinical symptoms. The recovered patients recruited to our study should meet three criteria: (1) the recovered patients were infected only once during the past 12 month. (2) The recovered patients should be detected every month in the past 12 months by RT-PCR, and the result for every detection was negative for SARS-CoV-2 RNA, and two consecutively RT-PCR tests negative for SARS-CoV-2 RNA more than 24 h before sampling. (3) The recovered patients have no antibiotic therapy in the past 6 months. All the recovered patients were fully recovered. Details regarding the PRs and HCs are shown in Table 1. All samples were collected by throat swab. The collection, transportation, storage and testing of all RP samples were performed in strict accordance with the instructions for highly pathogenic pathogens of type II following the prevention and control protocols for COVID-19 [19]. The Institutional Ethics Review Committee of Zhoushan Hospital, Zhoushan, China approved the protocols used for collection of samples from subjects with COVID-19 (approval number: 2020-003).

**Table 1:** Detailed information describing samples tested in this study

Cohort	Gender	Average age (years)	Months after recovery for sampling	Sample name	Initial clinical severity
Recovered patients (RPs)	Male (n = 17)	36.0	> 12 month	a1–a8, a49–a63	Mild
	Female (n = 6)				
Healthy controls (HCs)	Male (n = 14)	37.6	> 12 months	CK9–CK15, CK17, CK19–CK25, CK27–CK40	NA
	Female (n = 15)				

### DNA extraction and sequencing

Samples were inactivated at 56°C for 30 min before DNA extraction. Extraction of nucleic acids was performed using nucleic acid extraction kits (QIAGEN), after which the concentration and purity were measured using a Thermo NanoDrop One (ThermoFisher Scientific, MA, USA). Extracted DNA was subject to DNA library construction using a standard NEBNext® Ultra™ II DNA Library Prep Kit for Illumina® (New England Biolabs, MA, USA) according to the manufacturer’s instructions, after which index codes were added. DNA libraries were generated from PCR amplicons targeting the hypervariable V3–V4 regions of the bacterial 16S rRNA gene and the ITS2 region of the fungal ITS gene. The V3–V4 regions of the 16S rRNA gene and the ITS2 region of the ITS gene were amplified with the following primer pairs: 338F/806R (5’-ACTCCTACGGGAGGCAGCA-3’, 5’-GGACTACHVGGGTWTCTAAT-3’) and ITS2-2043R/ITS4R (5’-GCATCGATGAAGAACGCAGC-3’, 5’-TCCTCCGCTTATTGATATGC-3’). After quality assessment using a Qubit@2.0 Fluorometer (ThermoFisher Scientific, MA, USA), the library was sequenced on an Illumina NovaSeq 6000 platform and 250 bp paired-end reads were generated (Magigene Co., Ltd., Guangzhou, China). On average, we obtained 124,038 clean reads/sample for the bacterial microbiome and 76,227 clean reads/sample for the fungal microbiome.

### Bioinformatics analysis

Fastp (an ultra-fast all-in-one FASTQ preprocessor, version 0.14.1, https://github.com/OpenGene/fastp) was used to control the quality of the Raw Data by sliding window (-W 4-M 20). The primers were removed using the cutadapt software (https://github.com/marcelm/cutadapt/) to obtain the paired-end clean reads. Operational taxonomic units (OTUs) were clustered by the UPARSE pipeline [20]. The R and KRONA software were used to analyze the species community structure and phylogenetic relationship of different OTUs, respectively. The ANOSIM, MRPP, Adonis, and AMOVA functions of the Vegan and Pegas package in the R software were used to identify differences in community structure between groups and determine whether differences were significant.

Alpha diversity measurements for community diversity were calculated using QIIME2. Principal component analysis (PCA) was performed to obtain principal coordinates and evaluate the beta diversity. Sample cluster analysis was performed using the UPGMA (unweighted pair-group method with arithmetic mean) method. The function of bacterial communities was predicted based on the 16 S rRNA sequencing data using PICRUSt2 (phylogenetic investigation of communities by reconstruction of unobserved states) (https://huttenhower.sph.harvard.edu/picrust/).

### Statistical analysis

The alpha-diversity and bacterial β-diversity were the equivalents of within- and between-habitat diversity, respectively. And, they were calculated using QIIME. Differences between groups were evaluated based on the alpha diversity index using R. A student’s *t*-test or Mann Whitney test was used to identify statistical differences between groups. LDA effect size (LEfSe) analysis was used to identify biomarkers of each group based on the homogeneous OTU_table using the Wilcoxon rank sum test in the R software with correction through FDR. A *p* < 0.05 was considered significant. Common statistics and charts were generated using GraphPad Prism 8 (GraphPad Software, San Jose, CA, USA) and Origin 2019b (OriginLab Corporation, Northampton, MA, USA).

## Results

### Oral bacterial diversity of recovered patients with COVID-19

**Fig. 1:**
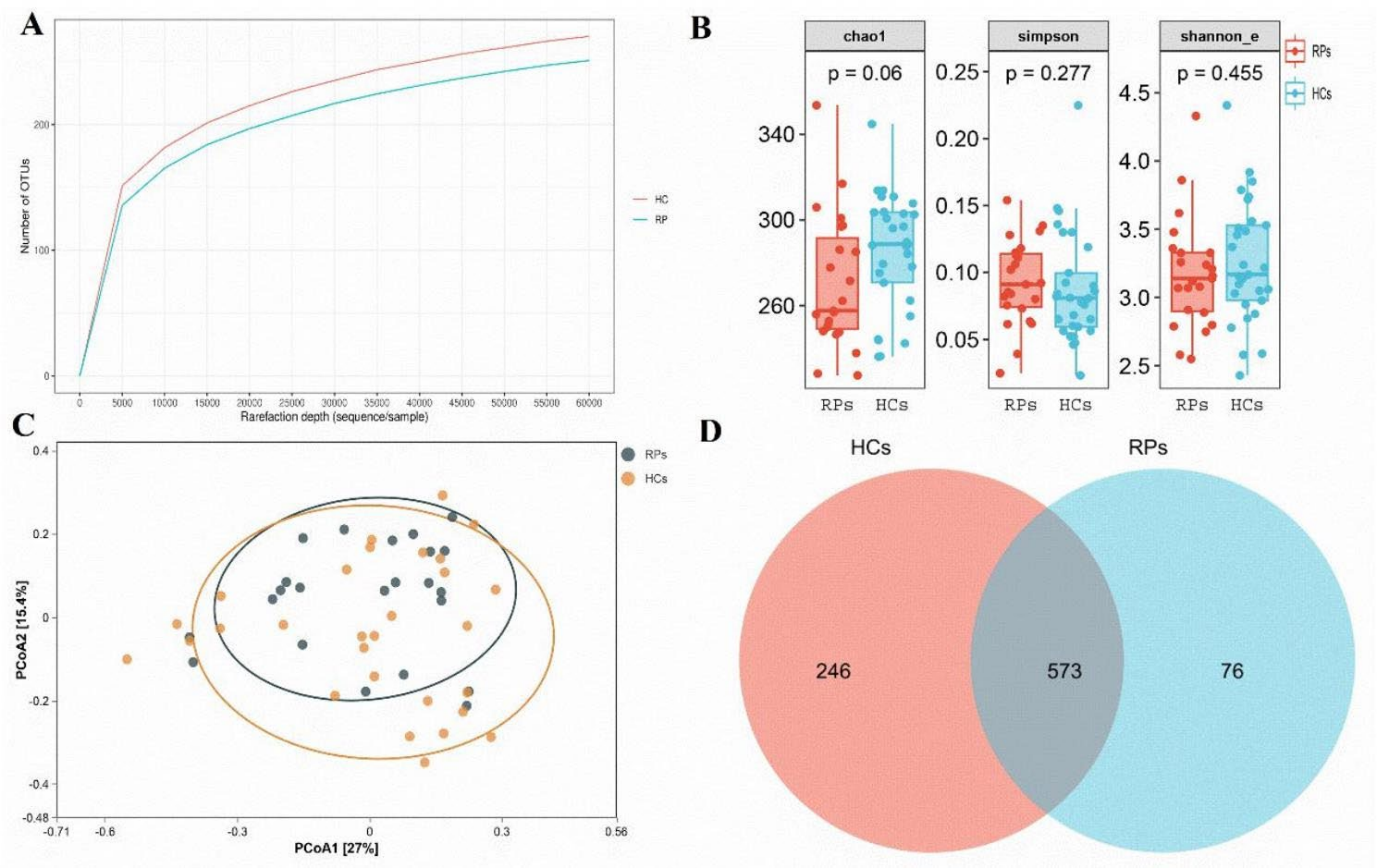
Oral bacterial microbial diversity of recovered patients and healthy controls. **(A)** Rarefaction curve comparing RPs (n = 23) and HCs (n = 29). **(B)** Alpha diversity boxplot of the oral microbial community based on the Chao 1, Simpson, and Shannon_e indexes. **(C)** PCoA based on Bray-Curtis similarity measures. **(D)**A Venn diagram displaying the overlaps between RPs and HCs. RPs: patients recovered from COVID-19, HCs: healthy controls

Rarefaction analysis showed that the OTU richness in each group approached saturation as the number of samples increased (Fig. 1A). Moreover, OTU richness was slightly decreased in RPs versus HCs. Evaluation of the alpha diversity revealed that oral microbial alpha diversity was slightly decreased in RPs versus HCs, but this difference was not statistically significant (Fig. 1A and Table S1). PCoA and NMDS analysis revealed no significant distinction in the beta diversity of oral microbial communities between groups (Fig. 1B C). A Venn diagram revealed that 573 of 895 OTUs were shared between groups, while 76 OTUs were sole to RPs (Fig. 1D). Overall, our results showed that the oral microbial diversity of RPs was similar to that of healthy controls. Other studies have shown that the oral microbial diversity was lower in confirmed patients with COVID-19 than healthy controls [21]. Therefore, the results of the present study indicated that oral bacterial diversity was restored as COVID-19 patients recovered.

### Phylogenetic profiles of oral bacteria in recovered patients with COVID-19

**Fig. 2:**
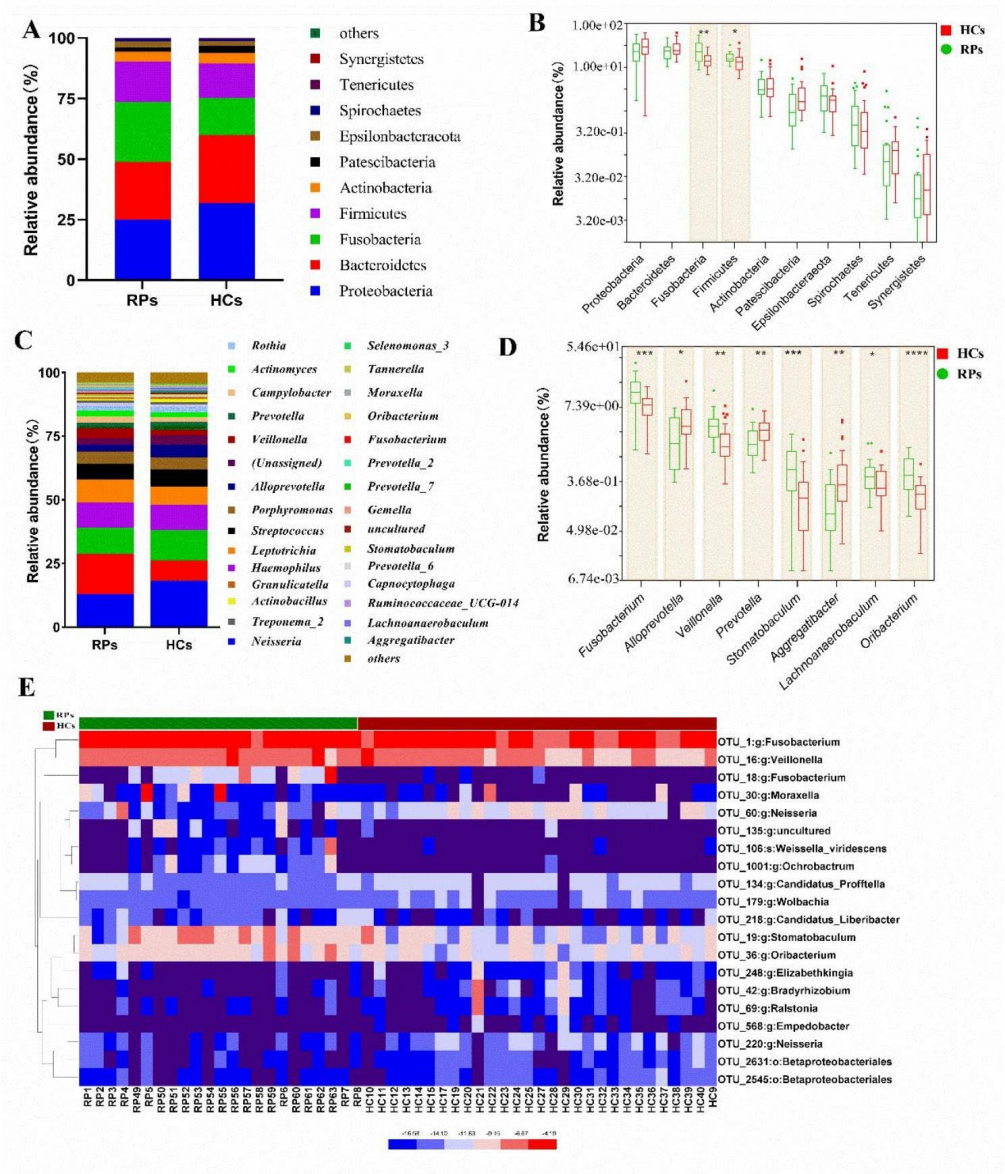
Bacterial community composition in RPs and HCs based on 16 S rRNA gene profiling. **(A)** Average compositions and relative abundances of bacteria at the phylum level for both groups. **(B)** Analysis of differences in phyla between RPs and HCs. **(C)** Average compositions and relative abundances of the bacterial community in both groups at the genus level. **(D)** Analysis of differences at the genus level between RPs and HCs. **(E)** Heatmap of the relative abundances of differential OTUs for each sample in both groups. *p < 0.05, **p < 0.01, ***p < 0.001

We further identified the bacterial composition and alterations in the oral bacterial microbiome in RPs and HCs. We found that the phyla Proteobacteria, Bacteroidota, Fusobacteriota, and Firmicutes accounted for 89.9% of sequences on average and comprised the four most abundant bacteria in RPs and HCs (Fig. 2A). Additionally, the phyla Fusobacteriota (*p* < 0.01) and Firmicutes (*p* < 0.05) were increased in RPs, compared with HCs (Fig. 2B).

At the genus level, six dominant bacteria, *Neisseria*, *Fusobacterium*, *Prevotella_7*, *Haemophilus*, *Leptotrichia*, and *Streptococcus*, accounted about for 62.9% of the total in both groups (Fig. 2C). The average composition and relative abundance of the oral bacterial microbiome at the genus level are shown in Fig. 2C and Table S2. Differential expression analysis using the Mann Whitney test revealed eight genera that differed between RPs and HCs (Fig. 2D). Among them, five (*Fusobacterium*, *Veillonella*, *Stomatobaculum*, *Lachnoanaerobaculum*, and *Oribacterium*) were significantly increased (*p* < 0.05), while three (*Alloprevotella*, *Prevotella*, and *Aggregatibacter*) were significantly reduced in RPs (*p* < 0.05) (Fig. 2D; Table S2). Furthermore, 20 OTUs were significantly increased or decreased in RPs versus HCs. Additionally, the heatmap showed that 10 OTUs were enriched in RPs, while another 10 OTUs were enriched in the HCs (Fig. 2E and Table S2). Overall, our results revealed a unique oral bacterial microbiota composition in patients that had recovered from COVID-19, which was characterized by similar bacterial diversity, but different bacterial abundances.

**Fig. 3:**
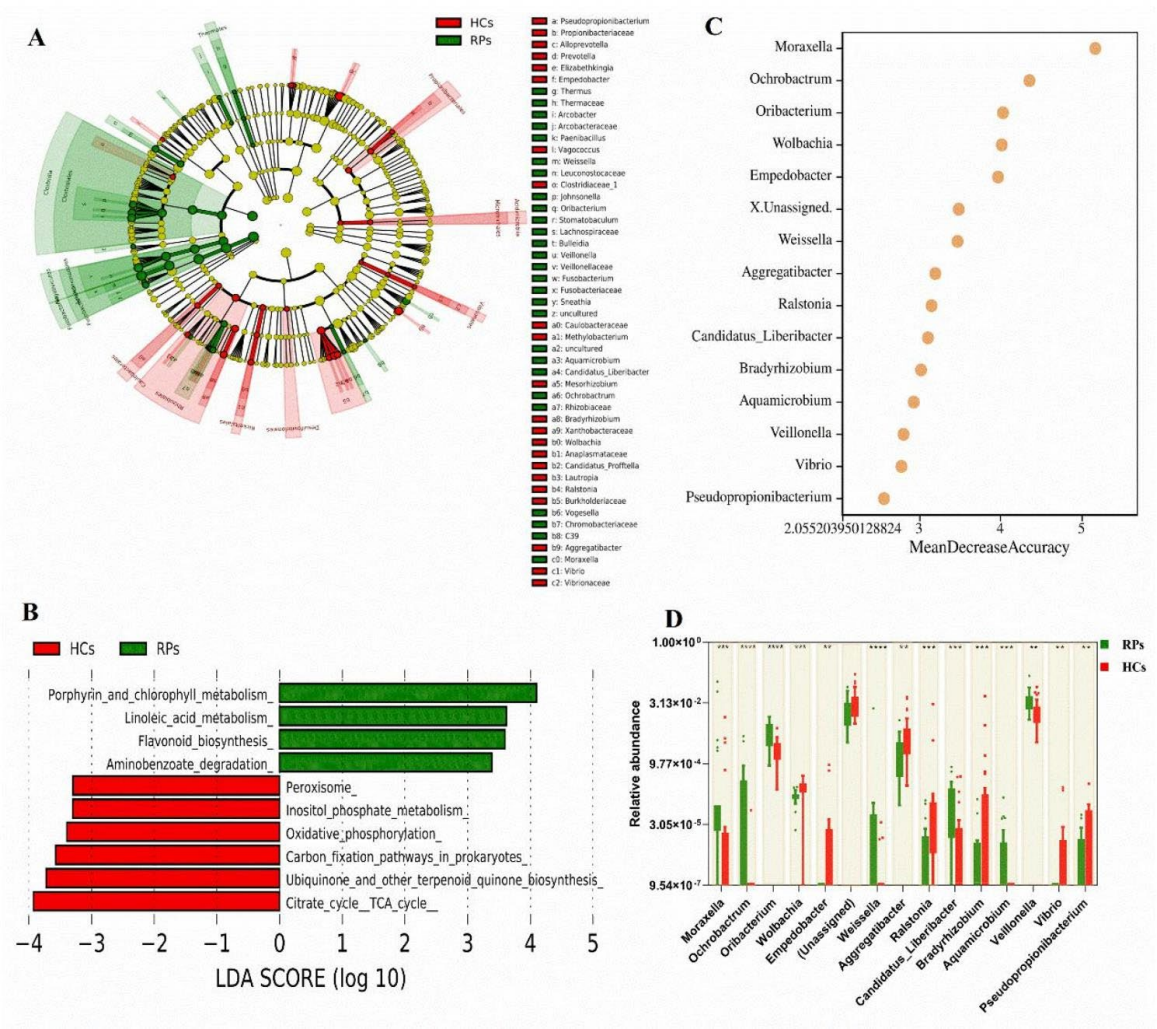
Screening of oral bacterial microbiome using LEfSe analysis and random forest analysis. **(A)** Cladogram of oral microbial structure and predominant bacteria revealed the greatest differences in taxa between RPs and HCs. **(B)** LEfSe analysis of the KEGG pathway identified a significant difference between RPs and HCs (LDA score > 2). LEfSe, linear discriminant analysis (LDA) effect size. LDA: linear discriminant analysis. KEGG: Kyoto Encyclopedia of Genes and Genomes. **(C)** Random forest plot showing the 15 most predictive bacterial genera that differentiate RPs from HCs. **(D)** Box plots of relative abundance of predictive bacterial genera and the *p* value were calculated using the Mann Whitney test. *p < 0.05, **p < 0.01, ***p < 0.001, ****p < 0.0001

To more specifically identify bacterial genera associated with recovery of COVID-19 patients, linear discriminant analysis (LDA) effect size (LEfSe) analysis was used to identify bacteria that differed significantly between groups. In this study, an LDA score of > 2 was used as the cut-off value. The cladogram representing the oral microbial structure and the predominant bacteria, showed the most differences in taxa between RPs and HCs (Fig. 3A). Functions were predicted using the copy number normalized OTU abundance table. In addition, we predicted the bacterial community function profiles using PICRUSt2 based on Kyoto Encyclopedia of Genes and Genomes (KEGG) orthologs and the KEGG pathway/module profile [22–24]. A total of 10 pathways that differed significantly between RPs and HCs were identified (Fig. 3B). Among these, four related to porphyrin and chlorophyll metabolism, linoleic acid metabolism, aminobenzoate degradation, and flavonoid biosynthesis, were remarkably increased, while six related to the citrate cycle, TCA cycle, and inositol phosphate metabolism, were under-represented in the RPs group.

To identify the featured oral bacterial microbes that differed between RPs and HCs, random forest analysis was performed. The 15 bacterial genera with the highest MDA values were shown in Fig. 3C. Among these, seven (*Moraxella*, *Ochrobactrum*, *Oribacterium*, *Weissella*, *Candidatus_Liberibacter*, *Veillonella* and *Aquamicrobium*) were more abundant in RPs, while the remaining eight were enriched in HCs (Fig. 3D).

### Structure of oral fungal communities in recovered COVID-19 patients

**Fig. 4:**
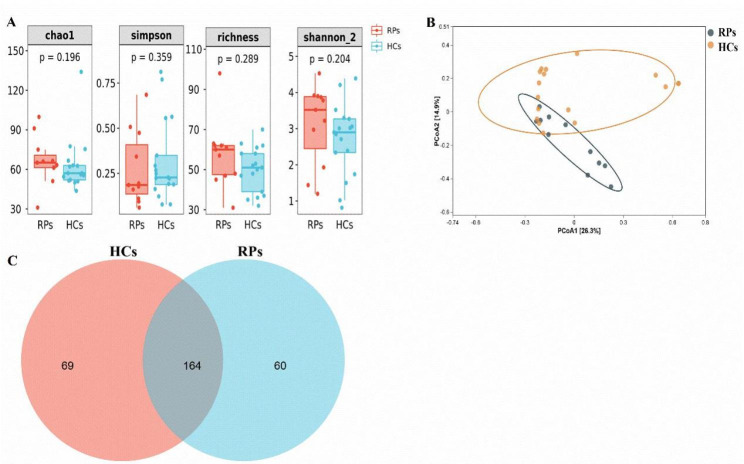
Oral fungal microbial diversity of recovered patients and healthy controls. **(A)** Alpha diversity boxplot in the oral fungal community based on the Chao 1, Simpson, Richness, and Shannon indexes. **(B)** PCoA based on Bray-Curtis similarity. **(C)** Venn diagram showing the shared and unique OTUs between groups. RPs: recovered COVID-19 patients, HCs: healthy controls

The fungal microbiome has recently gained recognition as a fundamental part of the human microbiome. Until recently, there has been a lack of research focusing on the oral mycobiome profiles of patients that have recovered from COVID-19. Thus, we analyzed 11 RPs and 17 HCs by ITS sequencing. The structures of the oral fungal communities were shown in Fig. 4A. The oral fungal communities did not differ significantly between RPs and HCs based on application of the Wilcoxon Rank Sum test to alpha diversity measurements (Fig. 4A and Table S3). Evaluation of the beta-diversity of the oral mycobiome at the baseline by PCoA, revealed no significant difference between RPs and HCs (Fig. 4B). A Venn diagram revealed that 164 of 293 OTUs were shared between groups, while 60 OTUs were found only in RPs (Fig. 4C).

### Phylogenetic profiles of oral mycobiome in recovered COVID-19 patients

**Fig. 5:**
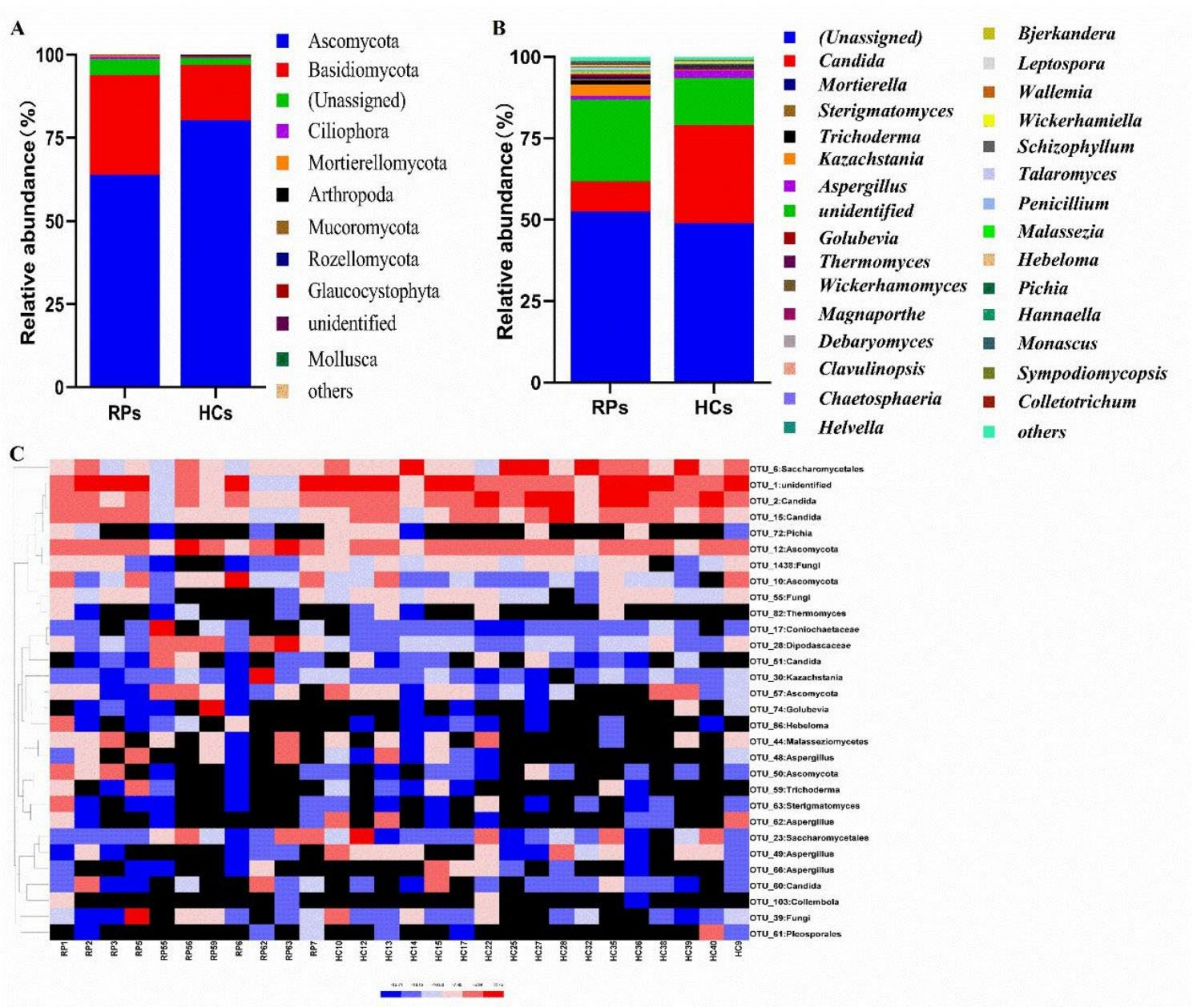
Fungal community composition of RPs and HCs based on ITS sequencing. Mycobiome composition at the phylum **(A)** and genus **(B)** level. **(C)** Heatmap of the relative abundances of differential OTUs for each sample in both groups

The relative abundances of members of the oral fungal community in RPs and HCs were shown in Fig. 5. At the phylum level, Ascomycota showed clear dominance in both RPs and HCs, with a mean relative abundance of 63.99% and 80.38%, respectively. The second most abundant phylum was Basidiomycota, which accounted for 29.85% and 16.51% of the total in RPs and HCs, respectively. The remaining phyla had relative abundances smaller than 5% (Fig. 5A and Table S4). Dominant genera were (Unassigned) (RPs 52.63% vs. HCs 48.91%), *Candida* (RPs 9.26% vs. HCs 30.16%), and unidentified (RPs 24.88% vs. HCs 14.39%) (Fig. 5B and Table S5).

**Fig. 6:**
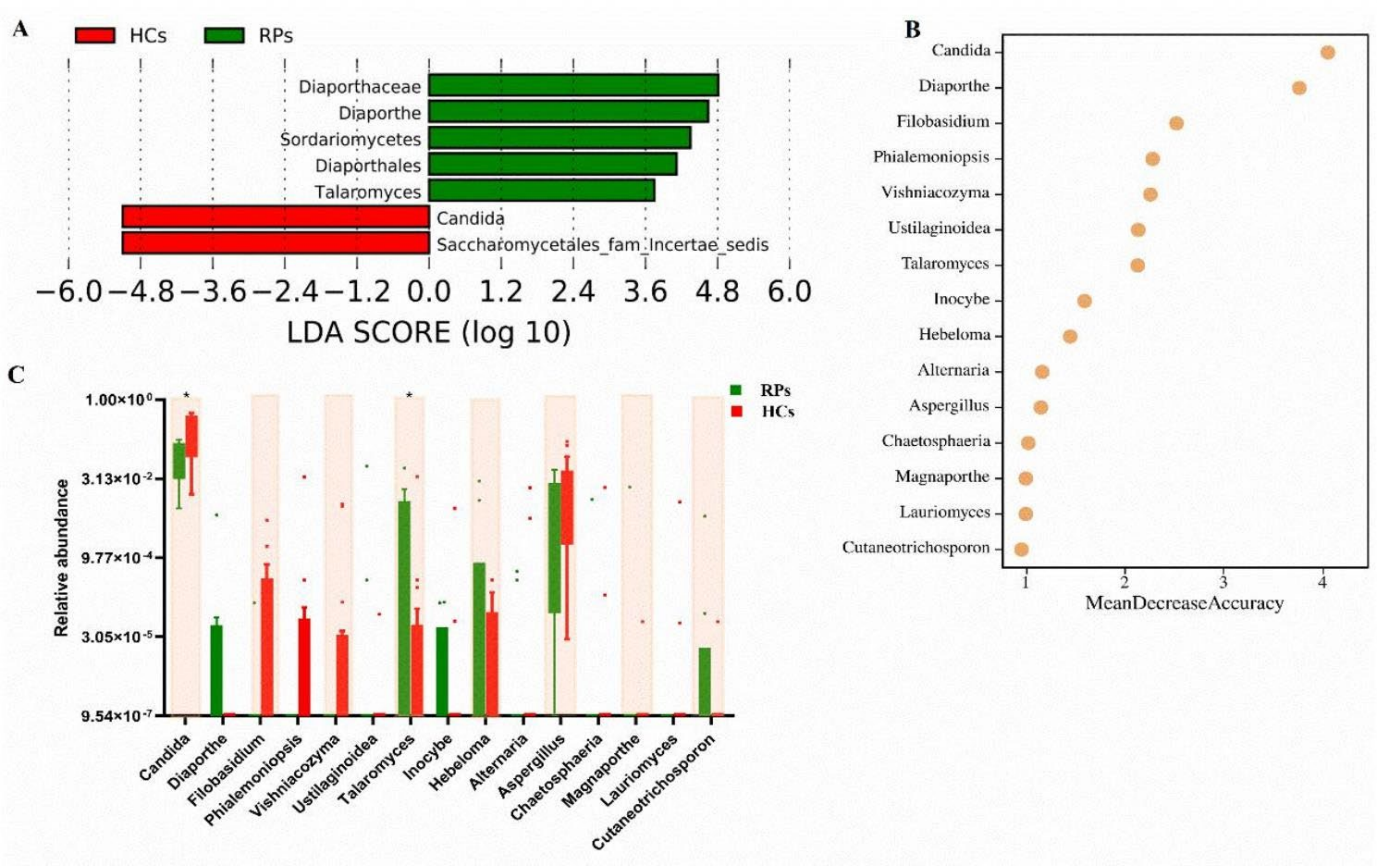
Screening of oral mycobiome by LEfSe analysis and random forest analysis. **(A)** LEfSe analysis of the classification units with significant differences between RPs and HCs (LDA score > 2). **(B)** Random forest plot showing the 15 most predictive fungi that differentiate RPs vs. HCs. **(C)** Box plots of relative abundance of predictive fungal genera. *p* values were calculated using the Mann Whitney test. *p < 0.05

LefSe analysis was used to compare microbial communities and identify specific oral fungi. Overall, seven differentially abundant fungal taxonomic clades were identified based on an LDA score > 2, among which five were significantly more abundant in RPs, and two were more abundant in HCs (Fig. 6A).

Furthermore, we performed random forest analysis to explore the potential for oral fungal microbes to discriminate RCs and HCs. The 15 fungal genera with the highest MDA values were shown in Fig. 6B. *Diaporthe*, *Talaromyces*, *Hebeloma*, and *Cutaneotrichosporon* were more abundant in RPs, while other genera were mainly enriched in HCs (Fig. 6C).

## Discussion

We compared the oral bacterial and fungal microbiomes of throat swab samples, obtained from recovered COVID-19 patients and healthy controls. A particularly profound finding was that both the oral bacterial and fungal microbiome of the recovered patients were restored to a large extent, but they did not completely return to normal. To our knowledge, this is the first study to characterize the oral bacterial microbiome and mycobiome in patients who have recovered for more than 12 months.

Previous studies have shown that oral bacterial microbial diversity was significantly decreased in confirmed COVID-19 patients versus healthy controls [21]. However, our data showed that the bacterial diversity was similar between RPs and HCs (Fig. 1). These results indicated that SARS-CoV-2 infection could induce oral microbial dysbiosis, but oral bacterial diversity was restored with recovery from COVID-19. Interestingly, Gao et al. found that the alpha diversity of confirmed cases and recovered patients was similar, but both were lower than those of healthy controls [25]. We speculated that this inconsistency was due to the different span of sample collection, and recovery of bacterial microbiome diversity appeared to occur slowly. However, we need to confirm this speculation by collecting more samples from patients at different stages of recovery. Our data showed that both the composition and abundance shifted remarkably (Figs. 2 and 3), while diversity remained similar (Fig. 1). We also found a significant increase in butyrate-producing *Fusobacterium* and a remarkable decrease in the oral opportunistic pathogen *Prevotella* in RPs relative to HCs (Fig. 2). Butyric acid plays an important anti-inflammatory role [26]. Therefore, the increase in butyrate-producing bacteria may be involved in the anti-inflammatory response during recovery from COVID-19. Previous studies have reported that *Prevotella* could drive inflammation, dampen innate immune responses [27], and contribute to oral inflammatory processes [28]. Abdul et al. found that the over-expressed *Prevotella* proteins could promote viral infection, suggesting that *Prevotella* plays an important role in in the progression of COVID-19 [29, 30]. Taken together, these findings indicated that the increase in butyrate-producing *Fusobacterium* and the decrease in the opportunistic pathogen *Prevotella* contribute to the recovery of SARS-CoV-2 infection.

This is the first study to the characterize the oral mycobiome of recovered patients with COVID-19. Previous studies have reported that higher fungal diversity was positively associated with HIV [31] and HBV/HCV infection progression [32], and a recent study showed that oral fungal diversity was increased in confirmed patients with COVID-19 [33]. However, our data showed similar diversity between RPs and HCs (Fig. 4). Taken together, these results indicated that fungal diversity gradually returned to normal during recovery from COVID-19, which was consistent with the trend of bacterial diversity observed in recovered COVID-19 patients.

The composition and potential microbiota marker species of fungal communities differed between RPs and HCs, although similar diversity was observed. Our findings showed that the specific opportunistic fungal pathogens *Aspergillus* and *Candida* were decreased in RPs (1.34% and 9.26%, respectively), compared with those in healthy controls (2.49% and 30.16%, respectively) (Fig. 5). A previous study showed that oral *Aspergillus* and *Candida* were significantly enriched in confirmed COVID-19 patients [33], but they did not follow up the recovered patients. Currently, it is not clear whether oral fungal dysbiosis is restored in recovered COVID-19 patients. Here, we provide the first evidence that oral fungal dysbiosis persists even after viral clearance. *Candida*, which is an opportunistic fungal pathogen, can cause oral candidiasis when the immune status is compromised, and candidiasis has been reported to be significantly associated with increased risk for COVID-19, as well as to cause complications related to oral infections [34, 35]. These results indicated that the decrease in specific opportunistic fungal pathogens such as *Candida* may contribute to the recovery of SARS-CoV-2 infection. Notably, the fungal community was not fully normalized, although it showed a recovery trend relative to confirmed COVID-19 patients. These findings suggest that long-term monitoring maybe necessary for patients recovered from COVID-19.

The human microbiome is closely related to recovery from a variety of diseases. As the second largest microbial community in humans, the oral microbiome plays crucial roles in maintaining oral homeostasis and is reportedly involved in the recovery of various diseases [36–38]. Our results suggested that oral bacteria and fungi may be involved in recovery from COVID-19. Based on the results of this study, there is potential for the application of microbial-assisted prognosis of COVID-19 patients. In addition, the results presented herein indicated that patients with SARS-CoV-2 infection should be followed from disease onset until after recovery, to thoroughly characterize the profile and the roles of the oral microbiome during SARS-CoV-2 infection.

Our study has several strengths. We first reported the characterization of the oral bacterial and fungal microbiome in patients recovered over 12 months from COVID-19. The previous study only reported several days follow-up of oral and gut microbiota in patients with COVID-19 [21]. Up to now, there was no reports of oral mycobiome in recovered patients of COVID-19. According to the previous study and our results, the particularly striking finding was that the oral microbiome including the bacterial and fungal characteristics of the recovered patients were only restored to some extent, but did not completely return to normal. Moreover, all recovered patients and healthy controls were from the same region, and the recovered patients had the same initial clinical symptoms. The key bacteria and fungus involved in recovery were also identified. Collectively, our study may inspire the researchers to better monitor and stratify the oral microbiome, as well as understand the key roles during the recovery of COVID-19. However, our present study has some limitations. To eliminate interference factors to the maximum extent, some subjects with a significant change in lifestyle and diet habits during one year follow-up, have been excluded in this study, therefore only 23 recovered patients were included at last. Due to the limitations of the actual situation, we included a small sample size with the patients only recovered over 12 months, but not patients recovered for more different time points. Large sample and more different time points verification are needed before clinical practice. Furthermore, it is an observational study and cannot elucidate the relationship of the oral microbiome and recovery process and recovery degree. It is also not certain whether similar alterations are observed in recovered patients who have different initial clinical symptoms and in different geographical regions. Uehara et al. found that the SARS-CoV-2 mRNA vaccine alters the oral microbiome, and vaccination may have beneficial effects on oral health [39]. While only unvaccinated subjects were recruited in our study, it is worth investigating whether vaccination accelerates recovery of oral microbiome in further study. With the research of further mechanism of microbiome affecting COVID-19, tracking oral microbiome and mycobiome changes maybe promising approach for the diagnosis and prognosis for COVID-19.

## Electronic supplementary material

Below is the link to the electronic supplementary material.

Supplementary Material 1

## Data Availability

The raw data supporting the conclusions of this article have been deposited in the NCBI database under the accession no SRP392877 (https://www.ncbi.nlm.nih.gov/bioproject/PRJNA869866) and will be made available by the authors upon request.
